# Supplementary material for: Comparative genomics provides new insights into the diversity, physiology, and sexuality of the only industrially exploited tremellomycete: Phaffia rhodozyma
Source: BMC Genomics. 2016 Nov 9;17:901. doi: 10.1186/s12864-016-3244-7 (PMC5103461; doi:10.1186/s12864-016-3244-7)
Supplement: Additional file 6: — List of orphan genes with links to PFAM (related to Additional file 1: Table S1). (ZIP 1428 kb) [file 12864_2016_3244_MOESM6_ESM.zip › BLAST_HTML_FTR/G05176_P.html]

BLAST Search Results


```
BLASTP 2.2.27+


Reference:
Stephen F. Altschul, Thomas L. Madden, Alejandro A. Schäffer,
Jinghui Zhang, Zheng Zhang, Webb Miller, and David J. Lipman (1997),
"Gapped BLAST and PSI-BLAST: a new generation of protein database
search programs", Nucleic Acids Res. 25:3389-3402.


Reference for
composition-based statistics:
Alejandro A. Schäffer, L. Aravind, Thomas L. Madden, Sergei
Shavirin, John L. Spouge, Yuri I. Wolf, Eugene V. Koonin, and
Stephen F. Altschul (2001), "Improving the accuracy of PSI-BLAST
protein database searches with composition-based statistics and
other refinements", Nucleic Acids Res. 29:2994-3005.


Database: nr
           71,551,133 sequences; 26,053,659,533 total letters


Query= G05176_P

Length=771
                                                                      Score     E
Sequences producing significant alignments:                          (Bits)  Value

emb|CED85599.1|  hypothetical protein [Xanthophyllomyces dendrorh...  1473    0.0  


 >emb|CED85599.1| hypothetical protein [Xanthophyllomyces dendrorhous]
Length=754

 Score = 1473 bits (3814),  Expect = 0.0, Method: Compositional matrix adjust.
 Identities = 742/742 (100%), Positives = 742/742 (100%), Gaps = 0/742 (0%)

Query  29   AMSKSSRSRARSNSINRESQISFDDGCEGYRTISRRPNDQLSDYQTGLAHNLSSFSINTL  88
            AMSKSSRSRARSNSINRESQISFDDGCEGYRTISRRPNDQLSDYQTGLAHNLSSFSINTL
Sbjct  13   AMSKSSRSRARSNSINRESQISFDDGCEGYRTISRRPNDQLSDYQTGLAHNLSSFSINTL  72

Query  89   ETFASIDDDKDNEDDALSSHYFLSRNDQSKSDQDFGAESDNGSSSKSSSLYKSSKRGKKP  148
            ETFASIDDDKDNEDDALSSHYFLSRNDQSKSDQDFGAESDNGSSSKSSSLYKSSKRGKKP
Sbjct  73   ETFASIDDDKDNEDDALSSHYFLSRNDQSKSDQDFGAESDNGSSSKSSSLYKSSKRGKKP  132

Query  149  SRLQSTLTGGSLKPFGSKPALVILDGLLGHESPSPPRARQEKKGKSKFISERRKNGPTIV  208
            SRLQSTLTGGSLKPFGSKPALVILDGLLGHESPSPPRARQEKKGKSKFISERRKNGPTIV
Sbjct  133  SRLQSTLTGGSLKPFGSKPALVILDGLLGHESPSPPRARQEKKGKSKFISERRKNGPTIV  192

Query  209  PPVPPVPEIHLSLDHSNSTLTSSYRARTNSTASWATTLDGSRTLSSIDLDSRPSRVDSQS  268
            PPVPPVPEIHLSLDHSNSTLTSSYRARTNSTASWATTLDGSRTLSSIDLDSRPSRVDSQS
Sbjct  193  PPVPPVPEIHLSLDHSNSTLTSSYRARTNSTASWATTLDGSRTLSSIDLDSRPSRVDSQS  252

Query  269  SRFALDDDYSMDEWESTLVPPSIGQSTSKHPSPPPRLRSLSLSQTAVHTTQPTFPRFPLT  328
            SRFALDDDYSMDEWESTLVPPSIGQSTSKHPSPPPRLRSLSLSQTAVHTTQPTFPRFPLT
Sbjct  253  SRFALDDDYSMDEWESTLVPPSIGQSTSKHPSPPPRLRSLSLSQTAVHTTQPTFPRFPLT  312

Query  329  PSPSATSSSSAYREEHPIGPIRSHSLSARDPSDSYLVASSTVSNSSCIDPKYPLPYPRHS  388
            PSPSATSSSSAYREEHPIGPIRSHSLSARDPSDSYLVASSTVSNSSCIDPKYPLPYPRHS
Sbjct  313  PSPSATSSSSAYREEHPIGPIRSHSLSARDPSDSYLVASSTVSNSSCIDPKYPLPYPRHS  372

Query  389  PQSPVFQPYDDHHYSQPPVIPPSPSPLPSPNTLEHLILPTPAPKIGRRISSKILTTLFFS  448
            PQSPVFQPYDDHHYSQPPVIPPSPSPLPSPNTLEHLILPTPAPKIGRRISSKILTTLFFS
Sbjct  373  PQSPVFQPYDDHHYSQPPVIPPSPSPLPSPNTLEHLILPTPAPKIGRRISSKILTTLFFS  432

Query  449  SSKAEPVPEMVGRTSSIADKGECKERRNMAKRTLPSPLEGGGGPGHHHTDSPKGSFFRKK  508
            SSKAEPVPEMVGRTSSIADKGECKERRNMAKRTLPSPLEGGGGPGHHHTDSPKGSFFRKK
Sbjct  433  SSKAEPVPEMVGRTSSIADKGECKERRNMAKRTLPSPLEGGGGPGHHHTDSPKGSFFRKK  492

Query  509  RTMYIERKFEKEKEDEGKNGMDKKREGLLLDRDEGDNELRRSYESDIEAVTAKLLPLFTL  568
            RTMYIERKFEKEKEDEGKNGMDKKREGLLLDRDEGDNELRRSYESDIEAVTAKLLPLFTL
Sbjct  493  RTMYIERKFEKEKEDEGKNGMDKKREGLLLDRDEGDNELRRSYESDIEAVTAKLLPLFTL  552

Query  569  PPRTSVVLEPPPVAFRPLRPSPCGSNASEPLFSTAATPAASTPAGLGRSYSLPIKTDRDP  628
            PPRTSVVLEPPPVAFRPLRPSPCGSNASEPLFSTAATPAASTPAGLGRSYSLPIKTDRDP
Sbjct  553  PPRTSVVLEPPPVAFRPLRPSPCGSNASEPLFSTAATPAASTPAGLGRSYSLPIKTDRDP  612

Query  629  SSDNQPAFINPFELLLPHLPVSPSCLPSPSPHKISSQKSPDRTHDFTNSVLCPSILELTP  688
            SSDNQPAFINPFELLLPHLPVSPSCLPSPSPHKISSQKSPDRTHDFTNSVLCPSILELTP
Sbjct  613  SSDNQPAFINPFELLLPHLPVSPSCLPSPSPHKISSQKSPDRTHDFTNSVLCPSILELTP  672

Query  689  VPNSPTPTIIPEVYSKMSRPLPSLPPRSPPREENTMAGERRLYQLGRSSSENSNLTNPAR  748
            VPNSPTPTIIPEVYSKMSRPLPSLPPRSPPREENTMAGERRLYQLGRSSSENSNLTNPAR
Sbjct  673  VPNSPTPTIIPEVYSKMSRPLPSLPPRSPPREENTMAGERRLYQLGRSSSENSNLTNPAR  732

Query  749  SSLESEGESLVLLYLSSSTLSD  770
            SSLESEGESLVLLYLSSSTLSD
Sbjct  733  SSLESEGESLVLLYLSSSTLSD  754


Lambda      K        H        a         alpha
   0.310    0.128    0.371    0.792     4.96 

Gapped
Lambda      K        H        a         alpha    sigma
   0.267   0.0410    0.140     1.90     42.6     43.6 

Effective search space used: 8982341984232


  Database: nr
    Posted date:  Sep 23, 2015 12:05 AM
  Number of letters in database: 26,053,659,533
  Number of sequences in database:  71,551,133


Matrix: BLOSUM62
Gap Penalties: Existence: 11, Extension: 1
Neighboring words threshold: 11
Window for multiple hits: 40
```
